# Plate Versus Intramedullary Nail Fixation for Distal Fibula Fractures: A Systematic Review and Meta-Analysis of Randomised Controlled Trials

**DOI:** 10.7759/cureus.90130

**Published:** 2025-08-15

**Authors:** Benjamin Cook, Neal H Patel, Sandeep K Nayar

**Affiliations:** 1 Trauma and Orthopaedic Surgery, University College Hospital, London, GBR

**Keywords:** ankle fracture management, distal fibula fracture, fibular nail, intramedullary nail fixation, open reduction and internal fixation with a plate (orif with plate)

## Abstract

Equipoise exists regarding implant choice for treating unstable distal fibula fractures. This study aimed to compare intramedullary nail (IMN) fixation and plate fixation (PF), considering solely randomised controlled trials (RCTs).

A systematic review and meta-analysis of all published RCTs was conducted. The study protocol was registered with the International Prospective Register of Systematic Reviews (PROSPERO). The Medical Literature Analysis and Retrieval System Online (MEDLINE), Excerpta Medica database (EMBASE), and Cochrane databases were searched. Outcomes were patient-reported functional measures, union rates, complications, revision rates, and cost-effectiveness. The Cochrane risk-of-bias v2.0 tool was used for bias assessment.

Five RCTs involving 455 patients (222 with IMN and 233 with PF) were included. Patient ages ranged from 18 to 93 years, with a mean age of 56.4 years. Functional outcomes, measured by the American Orthopaedic Foot and Ankle Society (AOFAS) score and Olerud and Molander Ankle Score (OMS), showed no significant differences between IMN and PF at 24 months. Meta-analysis demonstrated no significant difference in OMS at one year between studies (mean difference -2.33, 95% CI -10.03 to 5.37, P=0.55). Complication and union rates were similar between the groups. One study suggested cost savings with IMN despite the higher initial implant cost. Some concerns were raised about the risk of bias assessment of all the included studies.

IMN and PF provide comparable functional recovery for distal fibula fractures with similar complication profiles. Patient-specific factors should guide the choice of fixation method. IMN may offer cost benefits for certain populations, though further research is needed. Larger longitudinal studies including outcomes such as return to work and sports are recommended to refine clinical guidelines for selecting the appropriate fixation method.

## Introduction and background

Ankle fractures represent a significant epidemiological burden, with an international incidence ranging from 107 to 187 per 100,000 individuals, making them one of the most common lower extremity fractures [1-3]. Given the increasing prevalence of fragility fractures in ageing populations, optimising management strategies is imperative.

Surgical management of ankle fractures continues to evolve, particularly in light of findings from studies such as the Ankle Injury Management (AIM) multicentre randomised controlled trial (RCT), which evaluated open reduction and internal fixation (ORIF) with non-operative management in a close contact cast (CCC) in patients over 60. This study highlighted the safety and cost-effectiveness of non-surgical treatment; however, they reported a 19% loss of reduction requiring conversion to ORIF and a 15% incidence of malunion in the CCC group, emphasising the need for refined surgical approaches in this population [4,5]. For unstable distal fibular fractures, ORIF with a lag screw and neutralisation plate or bridging plate via a lateral approach remains the standard treatment, offering reliable anatomical restoration. However, this is associated with well-recognised complications, including wound dehiscence and infection [6,7]. Alternative approaches, such as the use of a posterolateral approach and an antiglide plate for certain fracture configurations, can mitigate these risks to some extent but introduce other concerns, such as peroneal tendon irritation [8].

Intramedullary nailing (IMN) of the fibula emerged at the turn of the millennium as a minimally invasive alternative to plate fixation (PF). The technique offers several theoretical advantages: smaller incisions may reduce wound-related complications, the nail is secured within the bone, minimising hardware prominence, and nails offer improved biomechanical strength. If IMN fixation provides comparable fracture stability while reducing complications, it could represent a favourable alternative to conventional plating. 

A previous systematic review by Tas et al. suggested that IMN fixation of the distal fibula resulted in fewer wound-related complications, implant removals, and non-unions compared with PF. However, this review incorporated both RCTs and observational studies, and since its publication, an additional two RCTs have been published. Therefore, a systematic review and meta-analysis focusing solely on level 1 evidence is warranted. 

This study aimed to compare IMN and PF techniques for distal fibula fractures, assessing functional outcomes, complications, revision rates, and cost-effectiveness. By synthesising high-quality evidence, this review seeks to guide surgical decision-making and optimise patient outcomes.

## Review

Methods

The study protocol was designed and registered prospectively on the International Prospective Register for Systematic Reviews (PROSPERO) database (registration number: CRD42024571018). It is reported according to the Preferred Reporting Items for Systematic Reviews and Meta-Analyses (PRISMA).

Eligibility criteria

Study Design 

Only RCTs were included. All other study designs were excluded. 

Participants 

The study covered adults (aged 18 or older) with acute, unstable ankle fractures involving the distal fibula warranting operative management. Fracture patterns could be unimalleolar, bimalleolar, or trimalleolar, with or without associated syndesmotic injury, provided fixation of the fibula was part of the surgical procedure. Inclusion was not restricted by classification to ensure consistency with the eligibility criteria of the included RCTs.

Intervention and Comparators

The intervention was the use of a fibular IMN. Comparators were any form of PF.

Outcomes 

The primary outcomes were patient-reported functional outcomes at one year. Secondary outcomes were fracture union, complication rate, rate of revision and cost-effectiveness.

Setting

No restrictions were placed on the setting of the study. 

Language 

No restrictions were placed on the language of the study.

Information sources

The Medical Literature Analysis and Retrieval System Online (MEDLINE), Excerpta Medica database (EMBASE), and Cochrane Library databases were searched on 17^th^ November 2023 to identify relevant studies.

Search strategy 

To increase sensitivity and heighten precision, the Cochrane Group RCT filters were used in the search strategy for each database. Additional search terms used were (fibula*.ti,ab) AND (nail.ti,ab) AND (plate.ti,ab OR osteosynthesis.ti,ab OR internal fixation.ti,ab). References from published systematic reviews investigating the same or similar topics were also manually searched for relevant studies. 

Selection process

All search results were combined and collected on EndNote 20 (Clarivate Plc, Philadelphia, PA). Duplicate articles were removed. Two independent reviewers (B.C. and N.P.) initially screened titles and abstracts, followed by full-text review. Disagreements between the reviewers were resolved with consensus from the senior author (S.K.N.).

Data collection process

Data were initially extracted onto Microsoft Excel (Microsoft Corp., Redmond, WA) using a standardised proforma. Review Manager (RevMan version 5.3, The Cochrane Collaboration, London, UK) was then used for data analysis.

Data items

Data extracted from each paper included the number of participants, age, gender, comorbidities, functional outcomes, complications, cost-effectiveness, and any other secondary outcomes measured by the study for each intervention. Mean and standard deviations were extracted for all outcome measures where possible. 

Risk of bias

Risk of bias was assessed by two independent reviewers (S.N. and N.P.) using the Cochrane Risk of Bias tool 2.0 (RoB 2).

Data synthesis

Where data could be appropriately pooled, a meta-analysis was conducted using the Mantel-Haenszel Random-Effects Model. Heterogeneity among studies was assessed using the I² statistic, with I² values greater than 40% indicating substantial heterogeneity. Statistical significance was defined as a P-value less than 0.05. In cases where meta-analysis was not feasible, continuous variables were described using mean values and standard deviation. Additionally, a narrative synthesis of the results was performed.

Results

Study Selection

**Figure 1:**
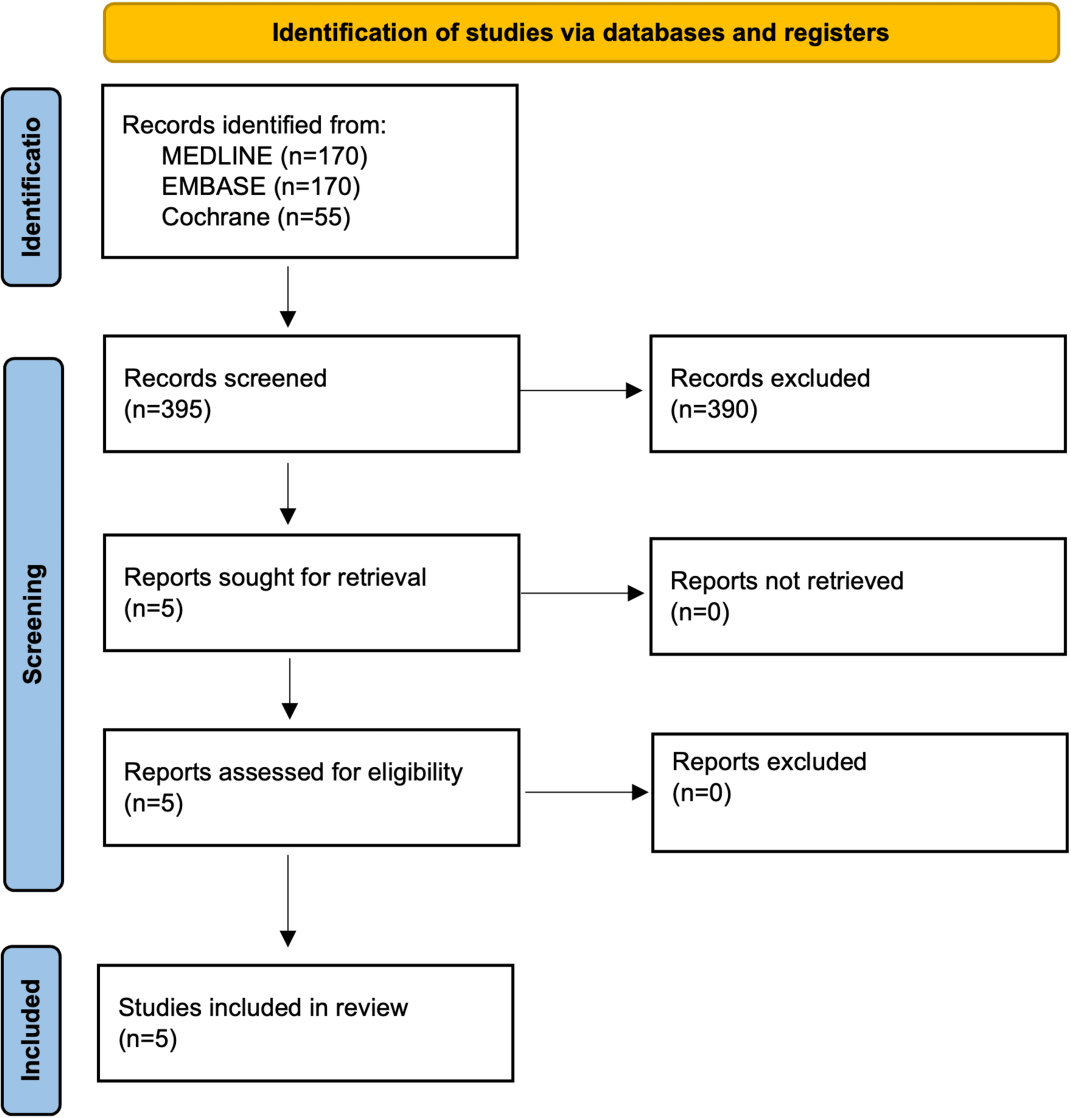
A PRISMA flow diagram outlining the study selection process PRISMA: Preferred Reporting Items for Systematic Reviews and Meta-Analyses; MEDLINE: Medical Literature Analysis and Retrieval System Online; EMBASE: Excerpta Medica database

A total of 395 articles were identified, of which five met the inclusion criteria and were retrieved for full-text review. All five articles were included in the meta-analysis. The PRISMA flow diagram is illustrated in Figure 1.

Study Characteristics

All five trials were published between 2014 and 2023 [9-13]. Study characteristics of each trial are reported in Table 1. All five studies looked at the use of fibula nail versus PF, which encompassed plates used in neutralisation, bridging or anti-gliding mode depending on fracture configuration. Sample size ranged from 51 to 125. Two of the trials were carried out at the same unit, looking at different study populations. 

**Table 1:** Study characteristics M: male; F: female; AOFAS: American Orthopaedic Foot and Ankle Society Ankle-Hindfoot score; OMS: Olerud and Molander Ankle score; VAS: Visual Analog Scale; MSK: Musculoskeletal; AO: Arbeitsgemeinschaft für Osteosynthesefragen (Association for the Study of Internal Fixation)

Primary author (year)	Mean age (range), years	M:F, n	Nail, n and the type used		Plate, n and the type used		Primary outcome(s)	Secondary outcome(s)
Stake et al. (2023) [11]	70	30:78	51	Acumed fibular nail	57	One-third tubular plate (DePuy Synthes), lateral distal fibula locking plate (DePuy Synthes), or Variax distal fibula plate (Stryker) was used in patients with poor bone quality.	AOFAS	Manchester-Oxford foot questionnaire, OMS, EuroQol five-dimension questionnaire, VAS for pain, complications, quality of reduction of fracture, non-union, and development of osteoarthritis
White et al. (2022) [9]	41.6 (18-64)	62:63	63	Acumed fibular nail	62	One-third tubular plate	OMS	Development of complications, requirement for reoperation, and assessment of reduction
Badenhorst et al. (2020) [13]	42.9	19:32	38	Acumed fibular nail	26	Acumed anatomical contoured plate	OMS, Grimby score	Swelling around the malleoli, plantar flexion, dorsiflexion, inversion, and eversion
White et al. (2016) [12]	74 (65-93)	25:75	50	Acumed fibular nail	50	One-third tubular plate (DePuy Synthes)	OMS	Incidence and nature of complications, Short MSK Functional assessment measure, appearance and comfort of scar measured by VAS
Asloum et al. (2014) [10]	53.3 (18-90)	37:34:00	36	Epifiza intramedullary nail (FH Orthopaedics)	35	AO reconstruction locking compression plate (DePuy Synthes)	Bone union	Complication rates, Kitoka score, OMS

Risk of Bias in Studies

All five included studies were found to have some concerns regarding their risk of bias. The majority of the concerns raised were due to deviations from the intended intervention and bias in the measurement of the outcome. One of the studies had further concern due to attrition bias, and another due to the selected patient demographic.

**Figure 2:**
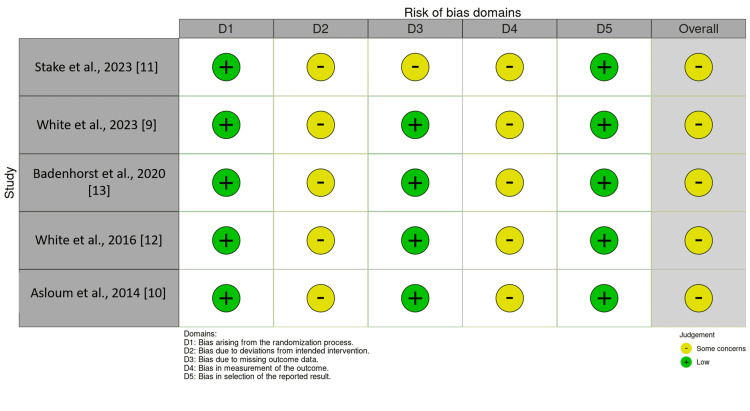
Risk of bias across individual studies using the Cochrane Risk of Bias tool 2.0 (RoB 2)

Results of Individual Studies

Patient demographics: A total of 455 patients were included in this meta-analysis. IMF nail fixation was performed in 222 individuals, and 233 were treated with PF. The follow-up ranged from six weeks to 24 months. The mean weighted age of the total study population was 56.39 years, with a range of 18 to 93 years. The male-to-female split was 173 to 282, and all five of the included studies were RCTs.

Inclusion and exclusion criteria: There were numerous similarities in the inclusion criteria across the different papers. Closed, isolated, and unstable or displaced fractures of the lateral malleolus were essential for inclusion in all five RCTs examined. Asloum et al. (2014) and Stake et al. (2023) additionally included patients who had sustained intertubercular and supratubercular bimalleolar fractures, as well as trimalleolar fractures, thus broadening the scope of applicability in these studies [9-11]. 

Time to surgery was specified in two of the trials; for inclusion, operative fixation must have occurred within three weeks of injury in the study by Stake et al. (2023) RCT and two weeks in the study by White et al. (2022) trial [11, 9].

Variation was observed in the age-specific inclusion criteria between studies. Two of the studies (White et al. 2016 and Stake et al. 2023) examined unstable ankle fractures in an elderly cohort, aged above 65 and 60 years old, respectively [12, 11]. Whereas White et al. (2022) selected for a relatively younger cohort, with patients only included if their age was between 18 and 65 years [9].

Patients were excluded if they had an associated fracture to the tibial pilon or talus in the study by Asloum et al. (2014) or if the fracture could be defined as pathological [10]. In Badenhorst et al.'s study (2020), they were excluded if they also had other injuries, if they had congenital abnormalities, or if they were deemed to have a fibular canal whose calibre could not physically accommodate a nail (smaller than 3.1 mm canal) [13]. Prior ipsilateral ankle injury or surgery excluded potential enrollees in studies by Badenhorst et al. (2020) and Stake et al. (2023), since this would likely reduce the ability of the studies to make accurate comparisons specifically between PF and IMN fixation in distal fibula fractures [13, 11].

Functional outcomes: Functional outcome scores were collected in all five of the RCTs. Stake et al. (2023) used the American Orthopaedic Foot and Ankle Society (AOFAS) Ankle-Hindfoot Score as a primary functional measure in patients >60 years old. The median AOFAS Ankle-Hindfoot Score at 24 months from surgery was found to be equivalent in the PF group and IMN group (95 vs 90, p=0.478). Other functional scores were included in this RCT, including the Manchester-Oxford Foot Questionnaire (MOxFQ) and the Olerud and Molander Ankle Score (OMS), which showed no significant difference between the two surgical groups at 24 months. Notably, at six months post-surgery, median AOFAS Ankle-Hindfoot Scores were significantly higher in the PF group compared to the IMN group (90 vs 83, p=0.023) in both per-protocol and intention-to-treat analyses. They also note that the post-operative median MOxFQ score at six months was higher in the PF group compared to the IMN group (14 vs 9, p=0.046) [11]. However, this finding did not show statistical significance in an as-treated analysis [11]. 

White et al. (2016) examined a similarly elderly cohort with patients aged >65 years, using the OMS to quantify functional outcomes post-operatively. They found that OMS fell from baseline after the fracture in both the PF and IMN groups and then recovered over a 12-month follow-up period with no difference in mean OMS at any stage. They note that although IMN produced a 3.6 times higher mean OMS at 12 months compared to PF, the trial would have needed seven times the number of patients in order to be adequately powered to show possible superiority in this functional outcome measure [12].

Asloum et al. (2014) examined functional outcomes as a secondary outcome, using Kitaoka and OMS at one year from fixation. The mean OMS and Kitaoka scores were significantly higher in the IMN group compared to the PF group (97 vs 83 for OMS; 96 vs 82 for Kitaoka score), and thus, they have concluded that in this cohort, IMN produced superior functional recovery when compared to PF at one year [10].

Badenhorst et al. (2020) compared the OMS between the two cohorts at three, six, and 12 months and found no significant difference at any time point during follow-up. They also examined the Grimby score to evaluate the activity of the study population during the same follow-up timepoints, again finding no significant difference and concluding in their study population that PF and IMN are similarly effective at restoring activity and facilitating functional recovery from these types of fracture [13].

White et al. (2022) also compared the mean OMS during follow-up between the two groups. They found that although patients had significant improvement in OMS from three months to one and two years (55, 80, and 88, respectively, p<0.001), there was no statistical or clinical difference between the two groups in OMS at any time point after surgery when analysed on both a per-protocol and as-treated analysis [9].

Cost-effectiveness: Only one study attempted to provide an economic evaluation. White et al. (2016) compared PF to IMN in terms of cost, incorporating the upfront price of the implant alongside theatre running, antibiotic and dressing costs, as well as the cost of treating complications, whilst excluding initial hospital stay cost. They found that the initial cost of an implant for ORIF was £129 (for one-third tubular plate and seven screws), whereas an IMN with two locking screws was £549. However, when factoring in the cost of surgical outpatient consultations, days of hospital stay and dressing changes, an estimate of the totality of IMN treatment was £91 cheaper than PF (£865 versus £956). Furthermore, they go on to posit that this may be a conservative estimate - since days on the ward pre- and post-operatively were discounted in the analysis, and it may be suggested that these would be fewer in the IMN group; the IMN does not require the abating of trauma-related oedema prior to fixation, and patients are generally permitted to be fully weight-bearing after the operation [12].

Return to work/sport: Injuries in the RCT by Asloum et al. (2014) were stratified by mechanism, and a significant proportion of the total occurred in both work and sporting settings (8.45% and 25.53%, respectively) [10]. That said, none of the papers included return to work or sport as outcome measures, nor was the topic commented upon in the discussion. 

Complications: All five RCTs reported on complications. The two trials that focused on an elderly cohort reached contrasting conclusions in terms of complication rate between PF and IMN. Stake et al. (2023) found significantly more secondary complications, such as failure of fixation, symptomatic non-union and neurological complications in the IMN group compared to the PF group (24% vs. 14%, p = 0.205) in their cohort, which included a population aged >60 years old [11]. White et al. (2016) examined the incidence and nature of complications as a secondary outcome in their >65-year-old study population, and the only difference found was a significantly higher rate of wound infection after PF compared to IMN (16% vs 0%, p = 0.002) [12]. 

One study found that there were significantly fewer post-operative complications in the IMN group compared to the PF group (7% vs 45%, p=0.0014) when accounting for a broad range of adverse outcomes (skin necrosis, sepsis, secondary displacement and algodystrophy) [10].

One study examined scar size between the two groups and found that the IMN group had a significantly smaller median scar length compared to the PF group (1.5cm vs 10cm, p<0.001) [13]. This corresponds to the smaller incision sizes needed in the IMN technique, but they found this to be associated with no significant difference in infection rate at six weeks postoperatively.

White et al. (2022) reported no difference in the overall rate of complications or reinterventions between the IMN group and the PF group (28.6% vs. 29%, p = 0.955) [9].

Overall, one study of an elderly cohort found a significant difference in rates of postoperative complications, one study was neutral, and three studies were in favour of IMN over PF.

Union: All five RCTs reported on bone union, but only two studies examined it as a primary or secondary outcome measure. Asloum et al. (2014) found that one year after fixation, fracture union was achieved in 100% of the IMN group and 94% of the PF group, but that this was not a statistically significant difference (p=0.5605) [10]. Similarly, Stake et al. (2023) demonstrated no statistically significant difference in non-union rate at six months between PF and IMN (5% vs 8%, p=0.705) [11]. 

Two studies noted that both groups had 100% union rates at one year, with no loss of initial reduction and no cases of non-union identified [9,13].

One study reported one loss of reduction in the PF group, resulting in malunion, and one malunion in the IMN group; however, neither necessitated further surgical intervention. [12]

Results of Syntheses

**Figure 3:**
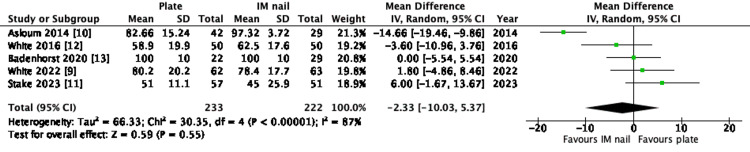
Forest plot of Olerud and Molander Ankle Score (OMS) one-year post-operatively IM: intramedullary

OMS at one year was reported in all five trials, allowing for pooled analysis. Overall, this showed no statistically significant difference in the patient-reported functional outcome measure between PF and IMN (mean difference -2.33, 95% CI -10.03 - 5.37, P=0.55). The forest plot of the meta-analysis results is illustrated in Figure 3.

Discussion

This systematic review and meta-analysis of level 1 evidence on PF versus IMN fixation for distal fibula fractures provides several key insights. Both PF and IMN fixation yield similar functional recovery outcomes, corresponding with previous reviews of both randomised and non-randomised studies [10]. Notably, no study extended follow-up beyond two years post-operatively.

In the most recent RCT, PF showed higher median AOFAS Ankle-Hindfoot scores and MOxFQ scores at six months in elderly patients, suggesting faster functional recovery with PF in this cohort [11]. However, this difference was not significant in the as-treated analysis, limiting its clinical relevance. Conversely, White et al. (2016) indicated that IMN fixation resulted in a higher mean OMS at 12 months in a comparably aged cohort, although a larger sample size would have been needed to confirm significance [12]. 

Financial analysis was limited but highlighted potential cost savings with IMN despite its higher initial implant cost. White et al. (2016) reported that IMN fixation was less financially burdensome than PF when considering overall treatment costs, attributed to fewer complications and shorter hospital stays commonly observed in elderly patients with IMN [12]. Further, financial analysis is warranted, given the high trauma burden of this type of fracture. 

Previous studies have suggested a reduced complication rate after IMN fixation compared with PF [10,12,14-16]. A recent systematic review deduced that the complication rate in patients treated with fibula IMN was lower than that with PF [17]. However, a subsequent RCT in elderly patients (≥60 years) reported significantly more secondary complications with IMN, predominantly symptomatic hardware and fixation failure [11]. The authors noted that their PF complication rate was low relative to the published [9], and their use of CT rather than radiographs to assess anatomical restoration may have increased the detection of fixation issues. Despite this, functional recovery was equivalent between groups across RCTs. In elderly patients, PF may confer advantages such as greater immediate construct stability in osteoporotic bone, improved control of fracture alignment, and reduced risk of fixation failure where intramedullary purchase is compromised by cortical thinning and a widened canal. Future designs, including next-generation headless screws that lock into the nail, may mitigate some of these concerns with IMN.

It is intuitive to conclude that IMN would carry a reduced risk of wound-related complications since, on average, PF requires an 8 cm skin incision, and IMN incisions range from 1 to 2 cm [18]. With clinical equipoise between the two techniques, this has been one area of clarity, and other literature suggests that for this reason, IMN may prove to be the superior option in patients with diabetic neuropathy or peripheral vascular disease, where wound healing is impaired and wound complications are more likely [19]. In these cases, the balance of risk/benefit shifts, and surgeons might choose to fix the ankle fracture in such a way as to minimise the risk of postoperative wound breakdown or infection.

The abovementioned needs to be achieved in the context of robust anatomical reduction and stabilisation. The present study found that both fixation techniques achieved equally effective stability for osseous union at the fracture site. This is important since poor fracture reduction is a proposed risk factor for the development of osteoarthritis (OA) [20]. Notably, the latest trial did not find any association between advanced OA at one- and two-years post-operatively, even in the context of the significantly worse reduction in the nail group compared to the plate group [11]. A nuanced approach should be taken, balancing the risk of poor wound healing with effective reduction, with consideration given to conversion to open reduction in suitable cases where a closed approach does not restore anatomy sufficiently.

Return to work and sport was an important area of study that was omitted from the trials. We cannot confidently extrapolate return to sport from the validated functional outcome scores used, given the unique and unpredictable insults encountered in this context. Kohler et al. (2023) carried out a biomechanical study looking at eight cadaveric specimens and demonstrated that in Weber C fractures, fixation with the IMN and two syndesmotic screws was significantly superior in withstanding rotational stress compared to the PF with two screws (p < 0.001; plate: 1.46 ± 0.33; nail: 1.80 ± 0.59; effect size (ES): 0.76) [14]. We may cautiously infer from this that a greater toleration of rotational force after unstable ankle fractures could translate to the sporting field and provide a more robust guarantee of long-term implant integrity in the context of large and unpredictable rotational loading. Further studies would be needed to examine the implications of this study on clinical practice, and examining return to work/sport could help illustrate functional recovery on a more qualitative basis, adding credibility to the quantitative functional scores that were widely used.

Limitations of this study include the small number of presently available RCTs and the heterogeneity between trials. Pooled analysis was only applicable to a single patient-reported outcome measure. Additionally, the trials did not include longitudinal outcomes beyond 24 months, limiting our certainty regarding long-term clinical efficacy. However, the study has several strengths. It focuses solely on level 1 evidence, thereby avoiding the confounding factors and statistical inaccuracies associated with non-randomised data. Since previous reviews, further RCTs have been published. It has been conducted in accordance with the PRISMA statement, minimising bias and providing a comprehensive evaluation while addressing methodological quality [21, 22, 23]. 

## Conclusions

This systematic review and meta-analysis of RCTs demonstrated that both IMN and PF provide comparable functional recovery from distal fibula fractures across adult and elderly populations while retaining a similar overall complication profile. However, specific patient factors should be taken into account, and clinical judgement must be applied to balance the risks and benefits. IMN may be more cost-effective for certain population groups, though further analysis is needed to confirm this. Longitudinal studies with larger study sample groups that include parameters such as return to work and sport would help clarify recommendations for selecting the appropriate device for different ankle fracture scenarios.
